# Rapidly Progressive Pauci-Immune Glomerulonephritis with Aberrant Fibrinoid Necrosis Associated with Atezolizumab, an Immune Check Point Inhibitor: A Case Report and Review of Literature

**DOI:** 10.3390/antib12010010

**Published:** 2023-01-25

**Authors:** Petros Nikolopoulos, George Liapis, Panagiotis Giannakopoulos, Ioannis Kotsantis, Konstantinos Drouzas, Sophia Lionaki

**Affiliations:** 1 2nd Department of Propaedeutic Internal Medicine, National and Kapodistrian University of Athens Medical School, Attikon Hospital, Section of Nephrology, 124 62 Athens, Greece; 2 1st Department of Pathology, National and Kapodistrian University of Athens, Laiko Hospital, 115 27 Athens, Greece; 3 2nd Department of Propaedeutic Internal Medicine, National and Kapodistrian University of Athens Medical School, Attikon Hospital, Section of Medical Oncology, 124 62 Athens, Greece

**Keywords:** acute kidney injury (AKI), vasculitis, immunotherapy, checkpoint inhibitors, immune related adverse effect, onconephrology

## Abstract

Stimulation of the antitumor activity of the immune system using immune checkpoint inhibitors (ICIs) has proven efficacy in the treatment of multiple types of cancer, inducing the speedily expanding approval of therapeutic indications for ICIs. The literature regarding the immune-related toxicities and nephrotoxicity of ICIs is limited. Herein, we present a patient with lung cancer treated with atezolizumab, an IgG1 monoclonal antibody aimed at the programmed death ligand 1 (PD-L1), who presented with vasculitic skin rash and rapidly deteriorating renal function, new onset of significant glomerular hematuria and proteinuria. The renal biopsy revealed acute necrotizing pauci-immune vasculitis, with fibrinoid necrosis. The patient received a course of high-dose glucocorticoids with recovery of renal function and skin lesions. Further immunosuppressive therapy was withheld, due to active malignancy in the lung, while oncology consultation recommended the continuation of treatment with atezolizumab, as the patient had shown substantial response.

## 1. Introduction

Immune checkpoint inhibition has been a considerable achievement of clinical oncology. The indications for immune checkpoint inhibitors (ICIs) have been progressively expanded, including treatments of various types of cancers. These agents act by blocking pathways called checkpoints, resulting in the modification of certain mechanisms of the immune system to regulate the immune response [1,2]. Immune checkpoint proteins are receptors based on the surface of cytotoxic T cells, interacting with their ligands, CD80/CD86, and their principal role is to protect the host against autoimmunity. These pathways can help malignant cells to skip cytotoxic T-cell–regulated death. Overexpression of these immunoregulatory molecules, including anti-programmed cell death protein 1 (PD-1) and anti-programmed cell death ligand 1 (PD-L1), is one of the pathways that cancer cells use to evade the immune system. Immune checkpoint inhibitors work by inhibiting the binding between receptor and ligands, overriding the suppression of cytotoxic T-cell recognition of cancer, and recursively succeed an effective anticancer immune response [3].

Atezolizumab is a humanized IgG1 monoclonal antibody targeting PD-L1 that has been indicated among others for the treatment of adult patients with locally advanced or metastatic urothelial carcinoma, as adjuvant treatment of non-small cell lung cancer and as first-line treatment of extensive-stage small cell lung cancer. Atezolizumab immune-related adverse events are hepatitis, colitis, pneumonitis, hypophysitis, hypothyroidism, and systemic inflammation [4,5]. Renal toxicity due to ICIs has been reported with nivolumab, ipilimumab, and pembrolizumab, but not with atezolizumab, except for a few cases of acute tubulo-interstitial nephritis [6,7].

## 2. Case Description

**Figure 1:**
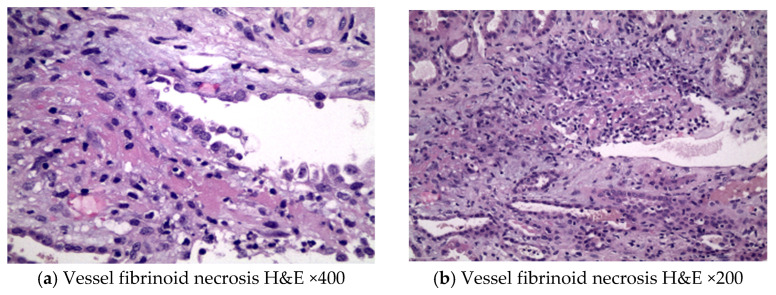
Fibrinoid necrosis in a parenchymal structure, most likely corresponding to a vessel wall. Although many sections were obtained, the exact vessel type cannot be clearly identified and determined (i.e., artery, vein, or lymph vessel), since it is entirely damaged ((**a**) H&E ×400, (**b**) H&E ×200).

**Figure 2:**
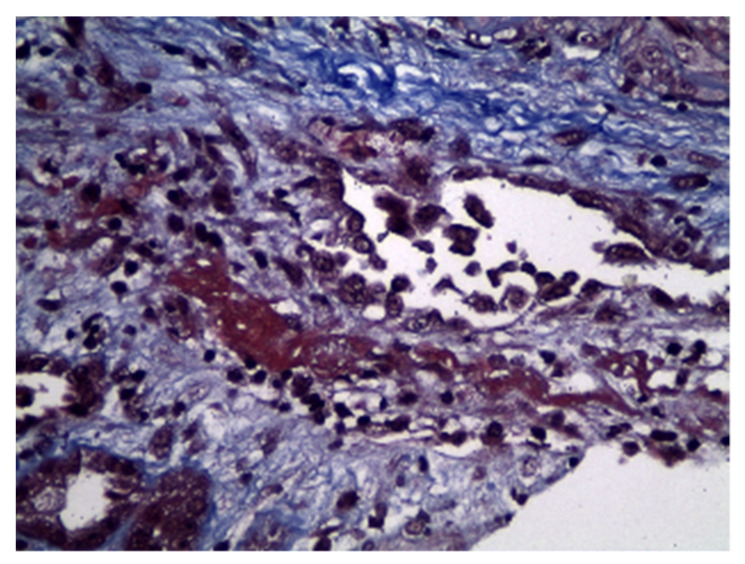
Fibrinoid necrosis is highlighted with a bright red (fuchsinophilic) color in Masson trichrome stain (Masson ×400).

A 73-year-old male presented to the emergency department due to purpuric skin lesions on lower limbs. At presentation he was also found to have new onset of acute renal dysfunction and microscopic hematuria of glomerular origin; phase-contrast microscopy of the urine sediment showed 80–100 dysmorphic RBC, non-nephrotic range proteinuria, and anemia. His past medical history was significant for hypertension, atrial fibrillation with a pacemaker for the last five years, extensive small cell lung cancer for the last 18 months under immunotherapy treatment, and immune-related pneumonitis grade 3, six months prior to presentation. Prior to admission, he was on flecainide, rivaroxaban, and atezolizumab. Physical examination revealed vasculitic skin lesions in the trunk and the lower limbs without edema of the lower extremities, which was developed within two weeks according to the patient’s report. His blood pressure was 137/75 mmHg and his heart rate was 80 bpm. Respiratory system examination revealed a decrease in respiratory wheeze. Laboratory examination showed hemoglobin of 8.2 g/dL and total leukocyte count of 8.900 per microliter with 76% polymorphs in the differential count. Biochemical parameters were as follows: blood sugar 100 mg/dL, serum creatinine 4.2 mg/dL, blood urea 151 mg/dL, and serum albumin 4.1 g/dL. Serum electrolytes and liver function tests were normal. Abdominal sonogram showed bilateral normal-sized kidneys with no pathological findings. Serological testing for anti-MPO-ANCA, anti-PR3-ANCA, anti-dsDNA, ANA, C3, and C4 was within normal limits. Imaging testing with a chest computed tomography showed a large concave lesion in the right upper lobe and consolidation of the pleural cavity, with reduced wall thickness of the lesion. Extensive emphysematous lesions and extensive fibrillar elements with confluence of the bronchial tubes of the lung were observed in both lungs as well as nodular lesions of the right lower lobe with indicative maximum cross-sectional diameter of 3.9 cm. The skin biopsy revealed T lymphocytes (CD4>CD8) with immunofluorescence (IF) negative for IgG, IgA, IgM, or C3, while histological findings of vasculitis were not observed. The renal biopsy included 18 glomeruli, with two of them being globally sclerotic (11% global glomerulosclerosis), and fibrinoid necrosis was documented (Figure 1a,b). Specifically, necrosis and inflammation developed in the wall of a vessel (Figure 2). Mesangium and the glomerular membranes were normal. The interstitial substrate showed mild to moderate alterations of interstitial fibrosis and tubular atrophy in ~25%. No glomerular crescents or glomerular necrosis were found (i.e., necrosis was limited to a vascular structure). By IF, no immune complex deposits were found; immunoglobulins and complement components were all negative. Taking all of the above in consideration, the diagnosis was consistent with acute necrotizing pauci-immune vasculitis with skin and renal involvement. Atezolizumab was temporarily discontinued, and immunosuppressive therapy with intravenous pulses of methylprednisolone (1000 mg daily for 3 days) was administered, followed by daily oral methyl-prednisolone (1 mg/kg/day) with gradual tapering. The patient responded to therapy: renal function gradually improved (serum creatinine 1.5 mg/dL, estimated GFR:59 mL/min/1.73 m^2^) and microscopic glomerular hematuria diminished to 10–15 RBC/hpf. Renal toxicity improved from grade 3 to grade 1 due to ESMO clinical practice guidelines for the management of toxicities from immunotherapies within two weeks of the initiation of treatment and the patient remains in remission of vasculitis with no need to administer an additional immunosuppressant agent [7].

## 3. Discussion

Acute kidney injury occurs in around 1–4% of patients treated with PD-L1/PD-1, and thus renal function should be evaluated prior to each infusion in order to withhold ICIS therapy in the event of significant renal dysfunction. Programmed death-ligand-1 is a transmembrane protein that is mainly expressed on activated T cells, B cells, macrophages, dendritic cells, monocytes, and in various cancer cells [8]. Programmed death-ligand-1/PD-1 interaction downregulates the immune response by inhibiting the activation and proliferation of T cells, resulting in a tolerance to endogen and external antigens. This mechanism of inactivation of the immune system and overexpression of PDL1 is used by many tumor cells. Inhibition of the PD-L1/PD-1 pathway results in T cells activation and an immune response against cancerous tissue [9]. As mentioned above, PD-L1 appears in tumor cells and in healthy tissue. Releasing the immune system activity against the tumor tissue by inhibiting the signaling pathways of PD-L1/PD-1 can produce multisystem adverse effects, and every organ has the possibility of being affected, including the skin, nerve system, endocrine system, lung, gastrointestinal tract, and kidney, which are the most frequently impacted by tubulointerstitial injury [10]. Renal tubule epithelial cells function as antigen-presenting cells for T cells provoking the immune response. It is known that renal tubule epithelial cells express major histocompatibility complex class II and modulate the immune response of T cells negatively [11]. Programmed death-ligand-1 is a negative costimulatory molecule that is not present in the glomerulus, thus the binding of the renal tubule epithelial cells PD-L1 to T cell PD-1 has a protective role against immune-mediated tubulointerstitial injury and side effects in general [12]. In contrast, anti-PD-L1 antibodies work by blocking PD-L1 receptor, which inhibits the activation of T-cells and attenuates their activity against antigens presented by renal tubule epithelial cells. T cells seem to play a major role in the pathogenesis of acute tubulointerstitial nephritis related to ICIs [13]. A different hypothesis has been developed regarding the interaction between lymphocytes and antigens in acute tubulointerstitial nephritis related to ICIs. Izzedine and colleagues [14] suggested that ICIs can activate specific inactive T cells, which have been sensitized by nephritogenic antigens, resulting in an activation of memory T cells against certain medications. Cortazar et al. [15] proposed that there could be a remodulation of the immune system, provoking the loss of acquired tolerance against self-antigens—a phenomenon that explains the period observed from the administration of the drug to the appearance of nephritis. Atezolizumab has been reported to produce adverse events. In a meta-analysis, among 751 patients, colitis appeared in 0.5%, hepatitis in 0.4%, pneumonitis in 0.8%, and hypothyroidism in 1.1% of the patients. The authors reported that 141 (15.6%) of the patients under atezolizumab therapy had arthralgias and 13.5% had musculoskeletal pain [16]. Wang et al. detected a 16.7% incidence of atezolizumab-related adverse events among 2015 patients using ICIs. That study also demonstrated a 2% rate of elevated aspartate and alanine aminotransferase and a 1% rate of pneumonitis [17]. Omar et al. reported ICIs-related acute kidney injury, which occurred relatively late after ICI initiation and was demonstrated by renal biopsy showing acute tubulointerstitial nephritis in most cases [18]. Other biopsy-proven glomerular pathologies associated with ICIs therapy include membranous nephropathy, cellular crescents with necrosis, granulomatous necrotizing vasculitis, and pauci-immune necrotizing glomerulonephritis in patients diagnosed with advanced solid or hematologic malignancy under ICIs therapy [18]. Han et al. identified the incidence of kidney injury in patients with ICIs therapy. In their systematic review, which included 27 randomized controlled trials and 15,063 patients with solid tumors who were following anti-PD-1/PD-L1 therapy with monoclonal antibodies in combination (or not) with chemotherapy, they demonstrated that ICIs can considerably increase nephrotoxicity in patients with solid tumors, in particular when combined with chemotherapy. The most common biopsy-proven diagnosis was acute interstitial nephritis [19]. Gallan et al. reported four patients with renal vasculitis or pauci-immune glomerulonephritis treated with nivolumab or pembrolizumab. Three patients had small- to medium-vessel vasculitis and one had focal crescentic pauci-immune glomerulonephritis [20]. In Table 1 are presented summarized the published reports of pauci immune glomerulonephritis and renal vasculitis due to immune check point inhibitors, the Antineutrophil cytoplasmic antibody (ANCA) serology, the initial steroid therapy, the use or not of other immunosuppressive agents and the resolution of AKI or the progression to chronic kidney disease.

AKI is not a rare complication in patients with malignancies, with the highest incidence is within the first year after diagnosis. The etiology of kidney injury includes treatment-related factors as well as comorbidities and patient’s related characteristics defining the AKI in cancer patients as multifactorial. Kidney injury in patients with malignancies, is related to a worsen prognosis and emerge the clinicians to interrupt and/or diminish dose of potentially beneficial active treatments and prolong the hospitalizations increasing mortality and morbidity of any cause. The continuous evolution and introduction of novel anti-tumor therapies and anticancer strategies is challenging and are expected to further improve patients’ outcomes but also new concerns are created regarding the safety, specificity and efficacy.Immune checkpoint inhibitors (ICIs) have transformed the landscape of oncology and have promoted the progression of anticancer therapies. Unfortunately, the antitumor immune response induced by ICIs can cause specific immune-related adverse events in almost every organ, system, including acute kidney injury (AKI). Approximately 22% of patients diagnosed with ICI-related AKI still need to continue or reuse ICI therapy in order to effectively treat or maintain cancer remission. Identification of other risk factors and recognize the patients at risk of developing AKI is important. Minimizing that risk with simple therapeutical strategies such as correction of hypovolaemia, cessation of nephrotoxic medications, monitoring of serum biochemistry and charting of fluid balance is the first step for protecting patients receiving ICI therapy, to develop AKI. Acute tubulointerstitial nephritis is the predominant lesion in biopsy proven AKI related to ICI therapy and proton pump inhibitors may predispose to acute tubulointerstitial injury and, must be discontinued in any patient with suspected AKI. Also, a prior history or simultaneous extrarenal immune-related adverse events, such as colitis, pneumonitis, dermatitis should raise suspicion for immune therapy related adverse event [25]. There aren’t established reliable clinical features to distinguish ICI-AKI from other potential etiologies The median time that AKI occurs after initiation of ICI medication is 14 to 16 weeks, but this period is variable from a few days for some patients to 1 year after initiation, for others. Sterile pyuria elevation of serum creatinine, subnephrotic-range proteinuria, are the most common findings, less common being microscopic hematuria of glomerular origin and/or oligoanuria but neither one is sensitive nor specific for AKI related to ICI therapy [26].

The median time to rechallenge with ICI after withdrawal is unknown. Switching ICI therapy simultaneously with use of low dose prednisone maybe effective but long-term outcomes are also unknown. An increasingly frequent cause of AKI related to ICIs in patients with cancer, poses diagnostic and management challenges to clinicians. Glomerular disease is less common than acute interstitial nephritis (AIN), but remains a serious adverse effect of ICIs. Researchers have reported various pathologies of glomerular disease associated with ICIs and mentioned the importance of kidney biopsy for diagnosing ICIs-induced kidney disease. The main therapeutic strategy is discontinuation of ICIs and glucocorticoid treatment that improves the renal outcomes. Accurate and early diagnosis of ICI related AKI, by kidney biopsy or other noninvasive biomarkers, the decision if or when to rechallenge a patient with ICI after ICI-related AKI, the identification of risk factors and the long-term outcomes of ICI related AKI are questions that need to be answered. Patients that develop AKI related to immunotherapy have in general favorable outcomes, with most of them, in some studies two third of patients, achieving kidney recovery. Early treatment with glucocorticoids is associated with a higher odds of kidney recovery [27]. The duration and dose of glucocorticoid therapy is debatable. A single-center study showed that tapering steroid therapy rapidly over a median of 20 days versus 38 days had equivalent outcomes [28]. Mycophenolate mofetil and infliximab are promising therapies alternative to steroid therapy for AKI due to ICI or for those cases of kidney injury from immunotherapy refractory to corticosteroids [27,28]. Larger studies are needed to determine and answer the above questions regarding the safety use of ICI therapies and the management of kidney injury related to them. The literature regarding ICIs-associated adverse events in the kidney is limited, and atezolizumab is one of the less studied medications. 

Here, we have reported a case of biopsy-proven acute necrotizing pauci-immune vasculitis affecting a vascular structure in the kidney, following therapy with atezolizumab for lung cancer. Importantly, no glomerular crescents or glomerular necrosis were found, since necrosis was limited to a vascular structure. The significance of this finding is not well understood and needs further exploration.

## Figures and Tables

**Table 1:** Published reports of pauci-immune glomerulonephritis and renal vasculitis associated with immune checkpoint inhibitor therapy.

Reference, Year	Immune Check Point Received	ANCA Serology	Initial Corticosteroid Therapy	Other Immunosuppressive Treatment	AKI Outcome
Cortazar et al. [15], 2020	Nivolumab	Negative	Corticosteroid, NOS	Rituximab	Full
Mamlouk et al. [18], 2019	Nivolumab	Negative	Prednisone 1 mg/kg	Rituximab	Complete
Mamlouk et al. [18], 2019	Ipilimumab plus nivolumab	Negative	Prednisone 1 mg/kg	Rituximab	Complete
Mamlouk et al. [18], 2019	Tremelimumab	NA	Methyl-prednisolone 2 mg/kg	Rituximab	Partial
Gallan et al. [20], 2019	Pembrolizumab	Negative	Pulse steroid, high-dose oral steroid	None	NA
Gallan et al. [20], 2019	Nivolumab	NA	NA	NA	NA
Gallan et al. [20], 2019	Nivolumab	Negative	Corticosteroid, NOS	None	Partial
Gallan et al. [20], 2019	Nivolumab	Negative	Pulse steroid, then oral steroid	IMCgp100	Complete
Cho et al. [21], 2018	Pembrolizumab	Positive	Corticosteroid, NOS	Cyclophosphamide	Partial
Lemoine et al. [22], 2019	Ipilimumab	Negative	Prednisone 1 mg/kg ×1 month, tapered over 4 weeks	None	Partial
Person et al. [23], 2020	Ipilimumab plus nivolumab	Negative	Methyl-prednisolone 200 mg IV daily	MMF	ESKD
Heo et al. [24], 2017	Pembrolizumab	Positive	Methyl-prednisolone 500 mg IV daily × 3 days, p.o taper	Cyclophosphamide	Partial

ANCA: antineutrophil cytoplasmic antibody; ESKD: end-stage kidney disease;; MMF: mycophenolate mofetil; NA: not available; NOS: not otherwise specified; IMCgp100: a bispecific biologic incorporating an engineered T cell receptor.

## Data Availability

Not applicable.
